# Applying machine learning in screening for Down Syndrome in both trimesters for diverse healthcare scenarios

**DOI:** 10.1016/j.heliyon.2024.e34476

**Published:** 2024-07-15

**Authors:** Huy D. Do, Jeroan J. Allison, Hoa L. Nguyen, Hai N. Phung, Cuong D. Tran, Giang M. Le, Trang T. Nguyen

**Affiliations:** a Hanoi Medical University, Hanoi, Viet Nam; b UMass Chan Medical School, Worcerster, MA, USA; c National Hospital of Obstetrics and Gynecology, Hanoi, Viet Nam; d Vietnam Academy of Military Science and Technology, Hanoi, Viet Nam

## Abstract

**Background:**

This paper describes the development of low-cost, effective, non-invasive machine learning-based prediction models for Down Syndrome in the first two trimesters of pregnancy in Vietnam. These models are adaptable to different situations with limited screening capacities at community-based healthcare facilities.

**Method:**

Ultrasound and biochemical testing alone and in combination, from both trimesters were employed to build prediction models based on k-Nearest Neighbor, Support Vector Machine, Random Forest, and Extreme Gradient Boosting algorithms.

**Results:**

A total of 7,076 pregnant women from a single site in Northern Vietnam were included, and 1,035 had a fetus with Down Syndrome. Combined ultrasound and biochemical testing were required to achieve the highest accuracy in trimester 2, while models based only on biochemical testing performed as well as models based on combined testing during trimester 1. In trimester 1, Extreme Gradient Boosting produced the best model with 94% accuracy and 88% AUC, while Support Vector Machine produced the best model in trimester 2 with 89% accuracy and 84% AUC.

**Conclusions:**

This study explored a range of machine learning models under different testing scenarios. Findings point to the potential feasibility of national screening, especially in settings without enough equipment and specialists, after additional model validation and fine tuning is performed.

## 1 Introduction

Down syndrome (DS) is a congenital defect caused by an extra 21^st^ chromosome. [1] From 2011 to 2015, Down Syndrome appeared in 8,031 annual live births, with an estimated 419,000 people living with the condition in Europe in 2015. [2] In the US, there were 6,756 annual live births with Down Syndrome, with a prevalence of 16.33 per 10,000 live births during 2016-2020. [3] There are no official reports on the prevalence of Down Syndrome in Vietnam. However, according to a study conducted in Da Nang on 14,335 live births from April 2015 to March 2016, this prevalence was 13.95 per 10,000. [4] Children with Down syndrome have a higher risk of congenital heart disease, deafness, ear infections, lung infections and autism leading to high mortality rates and reduced life expectancy. [5], [6], [7], [8], [9]

Currently, there are 3 methods of screening for Down syndrome in Vietnam: Non-Invasive Prenatal Testing (NIPT) based on chromosomal information from fetus's DNA in the mother's peripheral blood, the double biochemical test based on PAPP-A (Pregnancy Associated Plasma Protein A) and Free *β*-hCG in trimester 1, and the triple biochemical test which is based on AFP (Alpha Fetoprotein), hCG (Human Chorionic Gonadotropin) and uE3 (unconjugated Estriol) in trimester 2. Among them, NIPT has the highest sensitivity and specificity, both approaching 99%. [10], [11], [12] However, NIPT is more expensive than other screening methods (ranging from 3 to 6 million Vietnamese Dong) to be adopted as a universal screening program. The double test in trimester 1 and the triple test in trimester 2 have lower sensitivity and specificity, ranging from 50%-60% sensitivity and 85-90% specificity, and are being used widely due to their lower costs, about 400-500 thousand Vietnamese Dong, which is about 1/6 the cost of NIPT. [13], [14] Therefore, it would be ideal to develop a method that combines high sensitivity and specificity with a relatively lower cost, making it a viable candidate for inclusion in a universal screening program.

In recent years, the expansion of artificial intelligence (AI) to efficiently build decision support systems has led to new approaches to the early detection of Down syndrome. [15], [16], [17], [18], [19], [20] Machine learning is an important part of AI, and it gives computer systems the ability to learn automatically. Well-developed machine learning models have achieved sensitivities greater than 95% and higher with more data, higher than current double and triple testing methods currently being used in Vietnam. For example, Neocleous et al. developed a machine learning model that correctly identified all 129 cases of Down Syndrome in a total of 51,208 pregnancy cases. [21] Another deep learning model developed using nuchal ultrasound images achieved an AUC of 0.98 and 0.95 during training and testing, respectively. [20] Machine learning models can be implemented as a mobile app or a website, so prenatal screening using this method only requires a smart device that can access the software. Unlike NIPT, it is an inexpensive and easy-to-use method that can be used anywhere at any time. Therefore, this screening method could potentially be applied in the healthcare system in Vietnam, especially at the commune (community) level, where there are no trained specialists in genetics. This new method will help to increase the rate of pregnant women who can access the screening program and hopefully result in a reduced the frequency of undetected babies with Down syndrome.

There has been limited research on the application of machine learning in screening for Down syndrome in Vietnam. Therefore, it's necessary to conduct a study to develop such a model. To accommodate real-life settings, where there might be limitations in infrastructure leading to incomplete test results, which is common in Vietnam, this study was conducted to construct prediction models for Down Syndrome screening in first and second trimester using three distinct variable combinations and compare these models to find the most effective model in each trimester, seeking to maximize both sensitivity and specificity while requiring the least amount of information.

## 2 Method

### 2.1 Data collection

Vietnam National Hospital of Obstetrics and Gynecology is the largest leading hospital in north Vietnam specializing in obstetrics and gynecology. Thus, its patients come from all over the north Vietnam, make it a representative sample of the population. Data were collected from all satisfied medical records of pregnant women who visited the Vietnam National Hospital of Obstetrics and Gynecology from January 2012 and finished on December 2022. Eligible participants were pregnant women who had either or both ultrasound test results or prenatal screening test results (double or triple test), whether or not they had a history of miscarriage or stillbirth. Amniocentesis test results were also required to serve as a gold diagnostic standard for all participants. Those with multiple pregnancies or IVF pregnancies were excluded due to their differences in ultrasound and biochemical test results compared to those without these conditions.

A total of 16 variables were used to build machine learning models including two maternal characteristics: mother's age, history of having children with Down Syndrome; two double test indices (MoM-hcgb and MoM-papp-a); three triple test indices (MoM-ue3, MoM-afp and MoM-hcg); nine ultrasound test indices (gestational age, fetal crown-rump length, biparietal diameter, fetal heart rate, head circumference, abnormal nose (yes, no), abnormal fetal heart (yes, no), and femur length).

Ultrasound tests were conducted using Voluson E6 (GE, USA), Samsung HS60 (Samsung, Korea), and Samsung A80 (Samsung, Korea). Biochemical testing was done by Autodelfia (PerkinElmer, USA).

### 2.2 Building machine learning models

Four models were developed based on these approaches: K-nearest neighbor (KNN), Support Vector Machine (SVM), Random Forest (RF), and Extreme Gradient Boosting (XGBoost). All chosen algorithms are widely used and considered appropriate for classification problems in healthcare. [22], [23], [24], [25], [26], [27]

#### 2.2.1 Machine learning models included

• **K nearest neighbor (kNN)**: kNN algorithm, one of the simplest approaches, classifies observations into groups based on a “majority vote” of surrounding observations. The number of surrounding observations is determined by the value of k.
• **Support vector machine (SVM)**: SVM algorithms construct a hyperplane where the distance between groups of data points is at its maximum. This hyperplane is known as the decision boundary, separating the groups of data points on either side of the plane.
• **Random forest (RF)**: RF methods create multiple decision trees during a training phase. A final decision is based on the majority of the trees, and multiple independent decision trees are combined in parallel.
• **Extreme Gradient Boosting (XGBoost)**: XGBoost methods create output in the form of decision trees like that described for RF approaches. However, XGBoost methods combine results from the decision trees sequentially so that each new tree corrects the error of the previous tree, in contrast to the parallel RF approach.

Data were analyzed using Rstudio version 4.2.2. All machine learning models were built using caret package version 6.0-94.

### 2.3 Data processing

The initial dataset was cleaned and split into six subsets based on two trimesters and three different diagnostic approaches (ultrasound testing alone, biochemical testing alone, or both).

Table 1 provides an overview of variables included in each subset. Each of these variables affects or is affected by the presence of Down Syndrome in the corresponding trimester. For instance, older maternal age is associated with a higher chance of Down Syndrome, and children with Down Syndrome typically exhibit thicker nuchal translucency. These features were selected based on current literature and their availability in the initial dataset, reflecting their significance in clinical practice.

**Table 1:** Six variable subsets for analysis, Vietnam National Hospital of Obstetrics and Gynecology, 2012 - 2022.

	Trimester 1			Trimester 2		
	Ultrasound	Biochemical	Both	Ultrasound	Biochemical	Both
Mother's age	x		x	x		x
Fetus's age	x		x	x		x
History of having children with Down syndrome	x		x	x		x
Fetal crown-rump length	x					
Biparietal diameter	x		x	x		x
Head circumference	x		x	x		x
Fetal have nose	x		x	x		x
Fetal heart rate	x		x	x		x
Abnormal fetal heart				x		x
Fetal femur length				x		x
Nuchal translucency	x	x	x			
PAPP-A		x	x			
Beta-hCG		x	x			
AFP					x	x
hCG					x	x
uE3					x	x
Amniocentesis	x	x	x	x	x	x

No imputation methods were used for missing data. Selected cases in each subset should have all the corresponding variables value which means one case in trimester 1 biochemical subset must not have missing in any of the four variables nuchal translucency, *β*-hCG, PAPP-A and amniocentesis. Then, each of the four machine learning approaches was applied to each of the six data subsets, giving a total of 24 models to be trained and evaluated.

**Figure 1:**
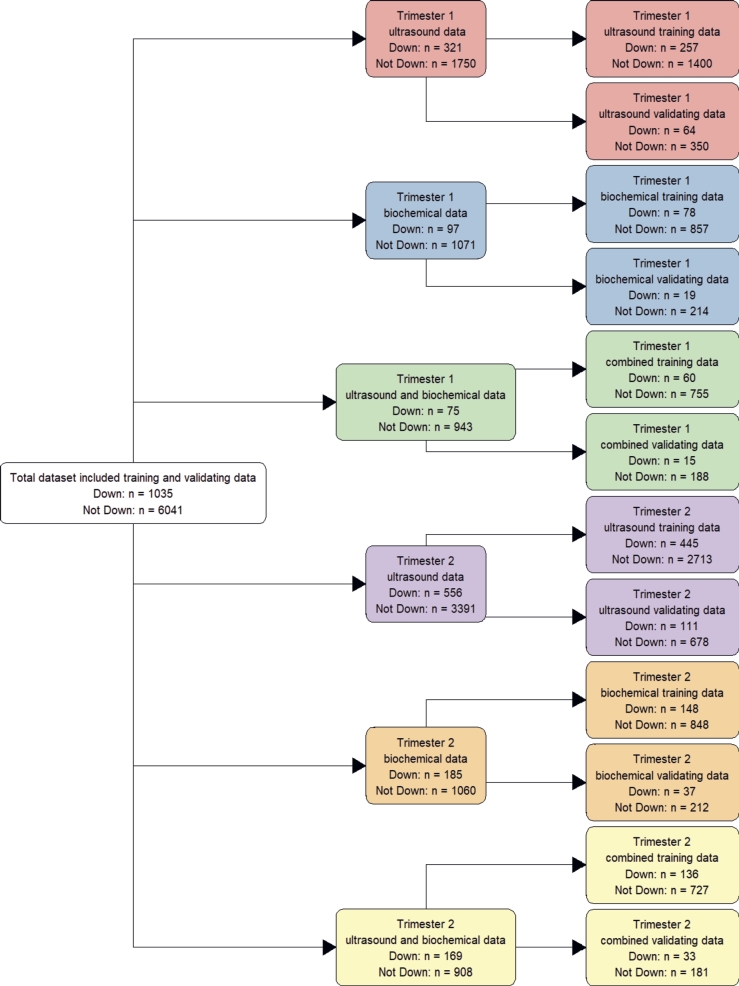
Overview of data subsets constructed for testing and validation, Vietnam National Hospital of Obstetrics and Gynecology, 2012 - 2022.

Fig. 1 presents an overview of the datasets and how the initial dataset was broken down into sub-datasets to build the different models. Selected cases in each dataset had all the corresponding variables. For example, each case in the trimester 1 biochemical dataset did not have missing data in any of the variables of nuchal translucency, *β*-hCG or PAPP-A. Each of these six datasets was divided into training sets for model training, and a validation set for model validation.

**Figure 2:**
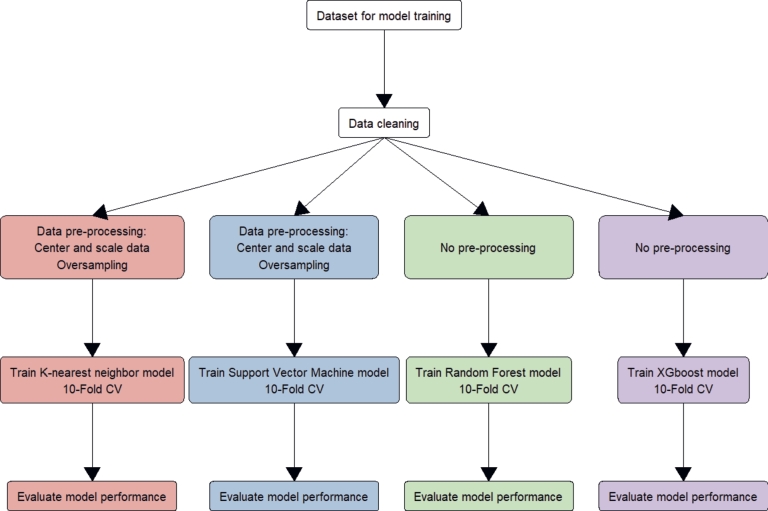
Training and evaluating machine learning models for each of six data subsets, Vietnam National Hospital of Obstetrics and Gynecology, 2012 - 2022.

The data were pre-processed by centering, scaling, and oversampling to balance the number of Down Syndrome cases with the number of non-Down Syndrome cases before training the kNN and SVM models. The RF and XGBoost models did not require any pre-processing steps. Each model was trained using 10-fold cross-validation (90% of data) and then validated (10% of data). Then, each of the 24 models were tested again using the validation set (Fig. 2).

#### 2.3.1 Fine tuning models

10-fold validation was chosen to search for the best hyper-parameters for all models with accuracy as the metric of choice. Accuracy is the popular learning metric in classification problem. During this process, a grid search strategy was used with the goal of minimizing the difference in accuracy between testing and validating results, keeping both at the highest value to reduce overfitting while maintaining performance. The hyper-parameters used to fine-tune each model included:

1. kNN: k is the number of nearest neighbors to consider when making a prediction or classification.
2. SVM: C controls the penalty for margin violations, which are data points that fall on the wrong side of the decision boundary or within the margin.
3. RF:
  • mtry controls the number of features (variables or predictors) randomly selected as candidates for splitting at each tree node during the tree-growing process.
  • ntree is the number of trees in the model.
  • max_depth (depth) is the maximum depth of tree.
4. XGBoost:
  • nrounds determines how many decision trees (weak learners) are sequentially added to the ensemble.
  • min_child_weight (mcw) controls the minimum sum of instance weight needed in a child.
  • subsample (ssp) controls the fraction of training data to be randomly sampled during each boosting round.
  • colsample_bytree (csb) determines the fraction of variables to be randomly sampled when building each tree.
  • max_depth (xgbdepth) sets the maximum depth of each tree in the ensemble.
  • eta (learning rate) controls the step size or learning rate used in the gradient boosting process.

The appendix provides a detailed process for training these machine learning models with information on each parameter (Table A.1, Table A.2, Table A.3). The complete code for all models can be found at the following link: https://github.com/Waltjer/Thesis-Paper-ShinyWebApp.

### 2.4 Assessing sensitivity, and specificity of machine learning models

The amniocentesis test served as the “gold standard” to diagnose Down Syndrome, and all other variables in each subset were used to predict it. The outcome was the probability of having Down Syndrome as predicted by the AI models; therefore, sensitivity (detection rate) and specificity (1 - false positive rate) were assessed across a range of thresholds and presented using Receiver Operating Characteristic (ROC) curves. For this study, true positives (TP) and true negatives (TN) were the correct predictions for patients' Down syndrome status, while false positives (FP) and false negatives (FN) were erroneous Down Syndrome predictions. A false positive was characterized as the prediction of a pregnant woman carrying a Down Syndrome fetus when she was not, whereas a false negative was characterized as the prediction of a pregnant woman not carrying a Down Syndrome fetus when she actually was. The positive predictive value (PPV) and negative predictive value (NPV) of these models were also calculated. The cut-off point for each test result used to classify whether a case had high or low risk of having Down Syndrome was chosen based on sensitivity and specificity. An optimal cut-off was defined as the cut-point that had the highest sensitivity and highest specificity based on the Youden method of the pROC package version 1.18.0.

### 2.5 Ethical issues

This research was funded by Vietnam 10.13039/100007225Ministry of Science and Technology and approved by the Institutional Review Board of the Vietnam National Hospital of Obstetrics and Gynecology, decision number 1042/CN-PSTW 24th December 2020. The research reported in this manuscript was supported by Fogarty International Center of the U.S. National Institutes of Health under a training grant “Training Program for Strengthening Research Capacity in Non-Communicable Diseases in Vietnam (TSORC-NCDs-VN)” [D43TW012188]. The content is solely the responsibility of the authors and does not necessarily represent the views of the funding agency. All data were entered into our web-based tool and stored there. Accounts to access the tool were provided to researchers on an as-needed basis.

## 3 Results

A total of 7076 pregnant women with the mean age of 31 were included in the study. Table 2 presents the characteristics of mothers and their fetuses according to the presence of Down syndrome. There were 1,035 pregnant women that had fetus with Down syndrome in both trimesters, 409 in trimester 1 and 626 in trimester 2. The mean age of pregnant women with Down Syndrome was 32.5 years, which was 1.8 years higher on average than those without a Down fetus. Fetuses with Down syndrome had a mean nuchal translucency thickness of 3.38, almost two times higher than the reference without Down Syndrome. Fetuses with Down syndrome also had lower mean PAPP-A, higher mean *β*-hCG in trimester 1, and higher mean AFP, lower mean uE3, and higher mean hCG in trimester 2. They also had shorter femurs than fetuses without Down Syndrome.

**Table 2:** Characteristics of mother and fetus according to the presence of Down syndrome, Vietnam National Hospital of Obstetrics and Gynecology, 2012 - 2022.

	Not Down	Down	Overall
	(N=6041)	(N=1035)	(N=7076)
**Mother's age (years)**			
Mean (SD)	30.7 (5.9)	32.5 (6.6)	31.0 (6.1)
Median (IQR)	30.0 (26.0, 35.0)	33.0 (27.0, 38.0)	30.0 (26.0, 36.0)

**Mother's age**			
>= 35	1679 (27.8%)	431 (41.6%)	2110 (29.8%)
< 35	4295 (71.1%)	604 (58.4%)	4899 (69.2%)

**Fetus's age (weeks)**			
Mean (SD)	114.0 (22.9)	108.1 (18.0)	113.1 (22.3)
Median (IQR)	114.0 (92.0, 129.0)	107.5 (93.0, 119.0)	113.0 (92.0, 127.0)

**History of having children with Down syndrome**			
	46 (0.8%)	11 (1.1%)	57 (0.8%)

**Fetal crown-rump length (mm)**			
Mean (SD)	62.0 (8.5)	65.0 (8.5)	62.5 (8.6)
Median (IQR)	61.0 (56.0, 68.0)	65.0 (59.0, 71.0)	62.0 (56.0, 69.0)

**Biparietal diameter (mm)**			
Mean (SD)	34.3 (11.9)	31.7 (9.9)	33.9 (11.6)
Median (IQR)	35.0 (23.0, 43.0)	32.0 (23.0, 38.0)	34.0 (23.0, 42.0)

**Head circumference (mm)**			
Mean (SD)	125.9 (43.9)	114.3 (34.6)	124.2 (42.8)
Median (IQR)	126.0 (83.0, 157.0)	115.0 (83.0, 137.0)	124.0 (83.0, 153.0)

**Abnormal fetal nose**			
	151 (2.5%)	45 (4.3%)	196 (2.8%)

**Fetal heart rate (beats per minute)**			
Mean (SD)	155.6 (9.5)	154.0 (10.0)	155.4 (9.6)
Median (IQR)	155.0 (149.0, 162.0)	154.0 (147.0, 160.0)	155.0 (149.0, 161.0)

**Abnormal fetal heart**			
	331 (8.3%)	35 (5.6%)	366 (7.9%)

**Nuchal translucency (mm)**			
Mean (SD)	1.9 (1.1)	3.4 (1.0)	2.2 (1.2)
Median (IQR)	1.5 (1.2, 2.6)	3.3 (2.8, 4.0)	1.7 (1.2, 3.1)

**PAPP-A (MoM)**			
Mean (SD)	0.7 (0.4)	0.5 (0.4)	0.7 (0.4)
Median (IQR)	0.7 (0.5, 1.0)	0.4 (0.3, 0.7)	0.7 (0.4, 1.0)

**Beta-hCG (MoM)**			
Mean (SD)	1.3 (0.8)	2.0 (0.9)	1.4 (0.8)
Median (IQR)	1.1 (0.7, 1.8)	1.8 (1.4, 2.8)	1.2 (0.7, 1.8)

**Fetal femur length (mm)**			
Mean (SD)	25.2 (7.7)	21.0 (6.1)	24.6 (7.7)
Median (IQR)	24.0 (19.0, 31.0)	20.0 (17.0, 24.0)	23.0 (19.0, 30.0)

**AFP (MoM)**			
Mean (SD)	0.8 (0.3)	0.8 (0.3)	0.8 (0.3)
Median (IQR)	0.8 (0.6, 1.0)	0.7 (0.6, 1.0)	0.8 (0.6, 1.0)

**hCG (MoM)**			
Mean (SD)	1.3 (0.8)	2.2 (0.9)	1.5 (0.9)
Median (IQR)	1.2 (0.8, 1.8)	2.1 (1.6, 2.8)	1.3 (0.8, 2.0)

**uE3 (MoM)**			
Mean (SD)	0.9 (0.4)	0.7 (0.3)	0.9 (0.4)
Median (IQR)	0.9 (0.6, 1.2)	0.7 (0.5, 0.9)	0.8 (0.6, 1.1)

In the validation process of trimester 1 (Table 3), the XGBoost model achieved the highest accuracy in all three datasets: 85% in ultrasound, 94% in biochemical, and 93% in the combined dataset. The XGBoost model based on only biochemical values showed equivalent accuracy with the XGBoost model based on the combined dataset. The random forest model had an accuracy of 94% for biochemical only testing.

**Table 3:** Model validation performance for trimester 1, Vietnam National Hospital of Obstetrics and Gynecology, 2012 - 2022.

Model	Threshold	Specificity	Sensitivity	PPV	NPV	Accuracy	TP	TN	FP	FN
**Ultrasound**										
k-nearest neighbor	0.44	0.81	0.80	0.44	0.96	0.81	51	284	66	13
Support Vector Machine	0.68	0.74	0.89	0.39	0.97	0.76	57	259	91	7
Random Forest	0.91	0.84	0.69	0.44	0.94	0.82	44	295	55	20
XGBoost	0.83	0.87	0.75	0.51	0.95	0.85	48	303	47	16

**Biochemical**										
k-nearest neighbor	0.28	0.91	0.63	0.38	0.97	0.88	12	194	20	7
Support Vector Machine	0.50	0.91	0.79	0.44	0.98	0.90	15	195	19	4
Random Forest	0.52	0.98	0.58	0.69	0.96	0.94	11	209	5	8
XGBoost	0.63	0.97	0.63	0.63	0.97	0.94	12	207	7	7

**Both ultrasound and biochemical**										
k-nearest neighbor	0.29	0.91	0.80	0.43	0.98	0.91	12	172	16	3
Support Vector Machine	0.54	0.85	0.93	0.33	0.99	0.85	14	159	29	1
Random Forest	0.91	0.84	0.80	0.29	0.98	0.84	12	158	30	3
XGBoost	0.91	0.94	0.73	0.50	0.98	0.93	11	177	11	4

*Note:* ROC = Receiver Operator Characteristics, PPV = Positive predictive value, NPV = Negative predictive value, TP = True positive, TN = True negative, FP = False positive, FN = False negative.

The highest accuracy in the trimester 2 validation process was achieved by the SVM model with a combined dataset, reaching 89% (Table 4). This was followed by XGBoost in the same dataset. In the biochemical dataset, XGBoost achieved the highest accuracy, while RF performed the best with the ultrasound dataset.

**Table 4:** Model validation performance for trimester 2, Vietnam National Hospital of Obstetrics and Gynecology, 2012 - 2022.

Model	Threshold	Specificity	Sensitivity	PPV	NPV	Accuracy	TP	TN	FP	FN
**Ultrasound**										
k-nearest neighbor	0.46	0.70	0.57	0.24	0.91	0.68	63	477	201	48
Support Vector Machine	0.55	0.59	0.75	0.23	0.93	0.61	83	401	277	28
Random Forest	0.77	0.79	0.46	0.27	0.90	0.75	51	538	140	60
XGBoost	0.85	0.74	0.54	0.25	0.91	0.71	60	499	179	51

**Biochemical**										
k-nearest neighbor	0.40	0.79	0.65	0.35	0.93	0.77	24	167	45	13
Support Vector Machine	0.57	0.69	0.76	0.30	0.94	0.70	28	146	66	9
Random Forest	0.81	0.79	0.59	0.33	0.92	0.76	22	167	45	15
XGBoost	0.67	0.86	0.62	0.43	0.93	0.82	23	182	30	14

**Both ultrasound and biochemical**										
k-nearest neighbor	0.34	0.87	0.64	0.47	0.93	0.83	21	157	24	12
Support Vector Machine	0.26	0.94	0.64	0.66	0.93	0.89	21	170	11	12
Random Forest	0.70	0.88	0.64	0.49	0.93	0.84	21	159	22	12
XGBoost	0.73	0.90	0.70	0.56	0.94	0.87	23	163	18	10

*Note:* ROC = Receiver Operator Characteristics, PPV = Positive predictive value, NPV = Negative predictive value, TP = True positive, TN = True negative, FP = False positive, FN = False negative.

**Figure 3:**
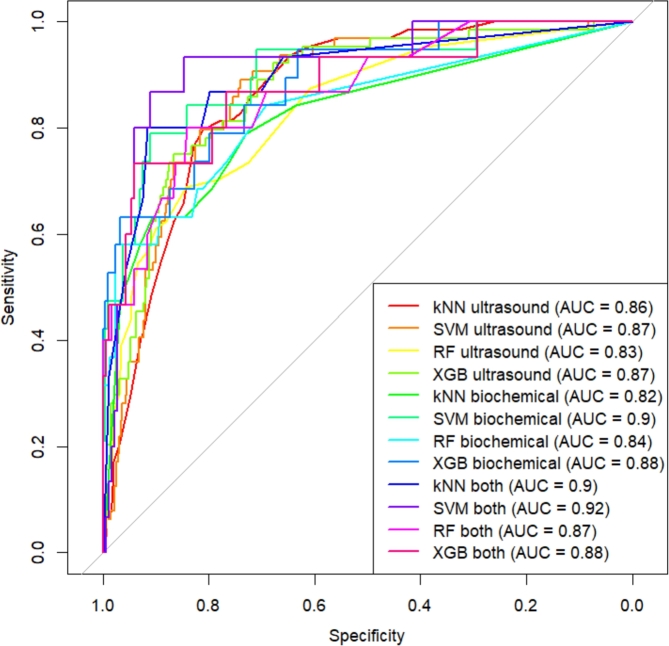
Receiver Operator Characteristics for machine learning models in trimester 1, Vietnam National Hospital of Obstetrics and Gynecology, 2012 - 2022.

**Figure 4:**
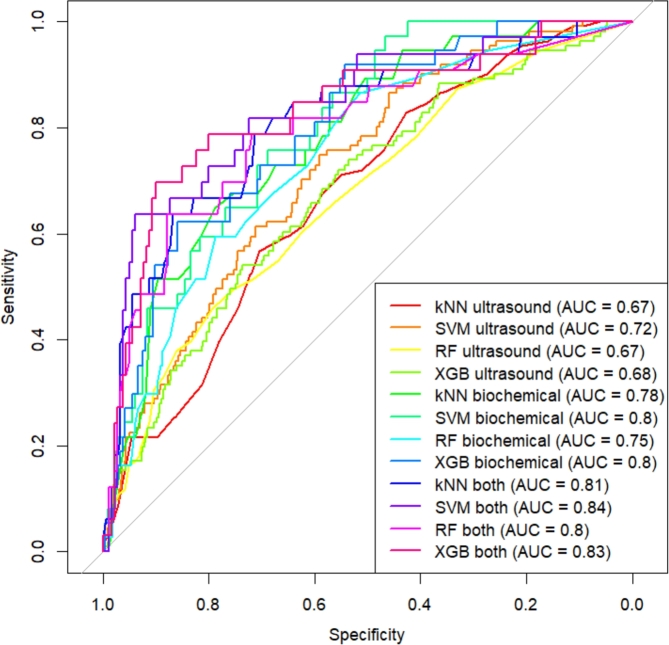
Receiver Operator Characteristics for machine learning models in trimester 1, Vietnam National Hospital of Obstetrics and Gynecology, 2012 - 2022.

Figure 3, Figure 4 show the ROC curves of all machine learning models in trimester 1 and trimester 2, respectively. The ROC values also show better performance in the combined set in both trimester, SVM models have the highest AUC in both trimester.

## 4 Discussion

### 4.1 Model accuracy

In trimester 1, the best models built on the combined dataset and the biochemical-only dataset showed equivalent performance. The model based on the ultrasound-only dataset had the poorest performance, suggesting that ultrasound data may not be as important in trimester 1. This suggests that the integration of biochemical data substantially enhances the accuracy of Down Syndrome screening during the early stages of pregnancy.

The superiority of the combined dataset model became even more evident in trimester 2, where it far outperformed the other two models. This reinforces the notion that a multi-faceted approach, encompassing both ultrasound and biochemical data, provides the most reliable screening results as pregnancy progresses, depending upon the trimester.

### 4.2 Trade-off between sensitivity and specificity

In screening for Down syndrome, sensitivity is as important as specificity. High sensitivity means that more cases can be detected through screening, while high specificity ensures that those classified as not having Down Syndrome really do not have the condition. In the case of high sensitivity, while having low specificity, more pregnant women will be classified as having a fetus with Down syndrome and require a confirmatory amniocentesis. This not only causes unnecessary psychological and financial impact for pregnant women and their families, but more importantly, it can also affect the fetus and the mother's reproductive health. Although amniocentesis is a confirmatory test and the gold standard, it is an invasive test with complications such as miscarriage, rupture of membranes, and infection. [28], [29] Thus, minimizing the number of pregnant women who undergo this test through ensuring high specificity is a requirement of Down screening methods. Based on these considerations, we choose the cut-off point with the highest accuracy to secure both high sensitivity and high specificity at the same time using the Youden method.

### 4.3 Comparison with current Down screening method

An international systematic review based on 56 studies, that included 204,759 pregnancies, of which 2,113 were affected by Down in trimester 1, showed the sensitivity of the best Double test to be around 68% while the best specificity would be around 95%. [30] Another study conducted by the same group of authors that included 59 studies involving 341,261 pregnancies with 1,994 cases of Down Syndrome in trimester 2 demonstrated similar results with 60-70% sensitivity and 95% specificity. [31] Our model with the highest accuracy in trimester 1 had better performance, with 73% sensitivity and 94% specificity, while the model with the highest accuracy in trimester 2 achieved 64% sensitivity and 94% specificity. Therefore, we concluded that our machine learning models had higher sensitivity and specificity than the current widely used Down screening methods, which are Double test and Triple test.

In comparison with NIPT, none of our models achieved 99% sensitivity nor specificity. However, our proposed method is still an inexpensive one that can be used as a universal screening method that can be applied in healthcare facilities with the capacity to perform either ultrasound or biochemical tests.

### 4.4 Application approach

In this study, we wanted to build and find the best and most suitable machine learning models customized to different testing capacities encountered across a range of facility types. Resources vary across the healthcare system, with some facilities being able to perform either the ultrasound test or the biochemical tests and some being able to perform both. By making models for a range of testing capacities, we can expand the scope of this screening program and let more pregnant women be screened for Down syndrome and inform the decision about amniocentesis testing for a more definitive diagnosis. Machine learning models are inexpensive yet effective and could be applied in healthcare facilities which don't have medical experts in prenatal screening.

The development of machine learning models using three distinct datasets - ultrasound only, biochemical only, and the combination of both - for Down Syndrome screening in both trimesters 1 and 2 presents a versatile and practical approach to address real-world healthcare scenarios. This approach is particularly relevant for healthcare facilities that may face limitations in conducting comprehensive screening tests, such as ultrasound or biochemical tests. By offering a choice of three models, each tailored to a specific dataset, this strategy allows healthcare providers to adapt their screening methods to match their available resources and patient needs effectively.

While the utilization of a combined model incorporating both ultrasound and biochemical data in trimester 1 is ideal when there's sufficient data from both tests, the option of relying solely on a model constructed from first-trimester biochemical data remains viable. This becomes especially relevant in scenarios where the healthcare facility lacks the capability to conduct Down's screening ultrasounds or when a patient opts not to undergo ultrasound testing. The accuracy of this standalone biochemical model closely aligns with that of the combined dataset model, offering a flexible choice of screening services. In the second trimester, it's essential to perform both types of assessments due to the clear difference in accuracy between the model constructed from the combined dataset and the model built using two datasets. The models created from these two datasets could be used as a point of comparison for the current screening methods.

In prenatal screening, early detection of problems with the fetus will lead to timely counseling, which is covered by health insurance. However, some pregnant women come to the clinic for their first prenatal examination in the second trimester for a variety of reasons. Therefore, a national screening system should cover both the first and second trimester.

These models can be integrated into the healthcare facility's administration program, which is the most convenient approach for every facility, or they can be developed as standalone mobile or web apps that patients can use at their own needs. Either way, due to their nature as mathematical models, they require minimal upfront hardware as well as maintenance and update costs. The greatest strength belongs to their easy-to-use aspect. Users only need to input data from the relevant trimester, and the software will calculate the risk of Down syndrome. Such low-cost, versatile, high-accuracy, and easy-to-use methods are suitable for low-resource settings, where the facility may lack the specialists and equipment to do either ultrasound or biochemical tests.

### 4.5 Strengths and limitations

This research represents the pioneering initiative in Vietnam to develop machine learning prediction models for Down Syndrome screening. Furthermore, it boasts an extensive dataset comprising over 1,000 cases involving pregnant women carrying fetuses with Down Syndrome. Additionally, this study introduced a diverse array of machine learning models, each comprised of specific inputs required during different trimesters. This multifaceted approach not only enhances options for pregnant women but also bolsters the healthcare system's utility, making it a valuable contribution to both patients and medical professionals.

Although the Vietnam National Hospital of Obstetrics and Gynecology is a major maternity hospital in the North with ethnic and regional diversity, the majority of cases were of the Kinh ethnic group and may not fully represent the entire Vietnamese population. Therefore, the initial machine learning models only “learned” from patients at a single site. In addition, some of the validation datasets had low numbers of Down Syndrome cases, limiting precision of the estimates and the ability to determine if observed difference were statistically significant.

## 5 Conclusions

For trimester 1, the accuracy of models based on combined testing or biochemical testing alone was more accurate than models based on ultrasound testing alone. For trimester 2, machine learning models based on combined ultrasound and biochemical testing produced higher accuracy than models based on either modality alone.

The machine learning approach that produced the highest accuracy varied by trimester and type of testing. For Trimester 1, XGBoost models produced ≥ 93% accuracy and 88% AUC for biochemical testing alone or combined testing. For trimester 2, support vector machine models produced 89% accuracy and 84% AUC for combined testing. The k-nearest neighbor approaches had the lowest accuracy in all settings.

Our findings suggest that screening for Down syndrome may be feasible in Vietnam using machine learning prediction algorithms based on currently available data and testing approaches in low-resource settings. However, these models should be applied at more hospitals to increase the generalizability and precision of the estimates and to refine the hyperparameters estimates before implementation. A range of new model types should be tested, and hyperparameters should be regularly updated with new and increasingly extensive data.

These models can be accessed via this Demo web app link.

## CRediT authorship contribution statement

**Huy D. Do:** Writing – original draft, Visualization, Validation, Software, Methodology, Investigation, Formal analysis, Data curation, Conceptualization. **Jeroan J. Allison:** Writing – review & editing, Methodology, Conceptualization. **Hoa L. Nguyen:** Writing – review & editing, Methodology, Conceptualization. **Hai N. Phung:** Methodology, Conceptualization. **Cuong D. Tran:** Supervision, Resources, Funding acquisition. **Giang M. Le:** Writing – review & editing, Supervision, Methodology. **Trang T. Nguyen:** Writing – review & editing, Supervision, Resources, Project administration, Methodology, Funding acquisition, Conceptualization.

## Declaration of Competing Interest

The authors declare the following financial interests/personal relationships which may be considered as potential competing interests: Huy D. Do reports financial support was provided by 10.13039/100007225Ministry of Science and Technology under grant number KC-4.0/19-25. Cuong D. Tran reports a relationship with Vietnam National Hospital of Obstetrics and Gynecology that includes: employment. If there are other authors, they declare that they have no known competing financial interests or personal relationships that could have appeared to influence the work reported in this paper.

## Figures and Tables

**Table A.1:** Hyper-parameters for each model, Vietnam National Hospital of Obstetrics and Gynecology, 2012 - 2022.

	kNN	SVM	RF			XGBoost					
	k	C	mtry	ntree	depth	nrounds	mcw	ssp	csb	xgbdepth	eta
**Trimester 1**											
Ultrasound	26	0.1000	1	50	3	200	2	0.6	0.6	5	0.10
Biochemical	9	0.0001	3	50	3	100	5	1.0	1.0	3	0.10
Combined	12	0.5000	2	200	3	200	1	0.6	0.6	5	0.10

**Trimester 2**											
Ultrasound	36	0.0200	5	50	3	200	5	1.0	0.6	7	0.10
Biochemical	59	0.4000	1	50	3	100	1	0.8	1.0	5	0.01
Combined	169	0.0100	7	200	3	50	1	1.0	0.6	3	0.10

**Table A.2:** Model training performance for trimester 1, Vietnam National Hospital of Obstetrics and Gynecology, 2012 - 2022.

Model	ROC	Sens	Spec	Accuracy
**Ultrasound**				
k-nearest neighbor	0.90	0.74	0.92	0.82
Support Vector Machine	0.85	0.78	0.83	0.80
Random Forest	0.82	1.00	0.02	0.85
XGBoost	0.83	0.93	0.33	0.84

**Biochemical**				
k-nearest neighbor	0.96	0.81	0.95	0.88
Support Vector Machine	0.92	0.77	0.77	0.77
Random Forest	0.88	0.98	0.50	0.94
XGBoost	0.92	0.99	0.41	0.94

**Both ultrasound and biochemical**				
k-nearest neighbor	0.99	0.82	0.99	0.91
Support Vector Machine	0.94	0.87	0.89	0.88
Random Forest	0.89	1.00	0.30	0.95
XGBoost	0.89	0.99	0.45	0.95

**Table A.3:** Model training performance for trimester 2, Vietnam National Hospital of Obstetrics and Gynecology, 2012 - 2022.

Model	ROC	Sens	Spec	Accuracy
**Ultrasound**				
k-nearest neighbor	0.80	0.63	0.80	0.72
Support Vector Machine	0.76	0.67	0.73	0.70
Random Forest	0.69	0.97	0.12	0.85
XGBoost	0.71	0.97	0.09	0.85

**Biochemical**				
k-nearest neighbor	0.85	0.65	0.89	0.77
Support Vector Machine	0.83	0.73	0.74	0.73
Random Forest	0.81	0.97	0.28	0.86
XGBoost	0.80	0.96	0.15	0.84

**Both ultrasound and biochemical**				
k-nearest neighbor	0.86	0.56	0.93	0.74
Support Vector Machine	0.86	0.78	0.81	0.79
Random Forest	0.82	0.97	0.32	0.87
XGBoost	0.83	0.98	0.23	0.86

## Data Availability

The authors do not have permission to share data.
